# The functional antibody landscape in HIV post-treatment controllers is heterogeneous

**DOI:** 10.1128/jvi.01790-25

**Published:** 2025-11-28

**Authors:** Nicholas E. Webb, Matthew J. Gorman, Lily J. Parker, Wonyeong Jung, Dansu Yuan, Jonathan Z. Li, Galit Alter, Boris Julg

**Affiliations:** 1 Ragon Institute of Mass General MIT and Harvard 200750 , Cambridge, Massachusetts, USA; 2 Brigham and Women’s Hospital, Harvard Medical School 1811 , Boston, Massachusetts, USA; Icahn School of Medicine at Mount Sinai, New York, USA

**Keywords:** system serology, antibody responses, HIV-1, post-treatment controllers

## Abstract

HIV-1 establishes a latent reservoir early in infection that persists during antiretroviral therapy (ART), typically leading to rapid viral rebound upon treatment interruption in most people living with HIV-1. However, a rare group known as post-treatment controllers (PTCs) can maintain low or undetectable viral loads for extended periods after ART cessation, offering critical insights into durable HIV remission. While immune responses are thought to play a key role in this control, the contribution of humoral immunity—particularly Fc-mediated antibody functions—remains insufficiently defined. In this study, we explored the influence of humoral responses on viral rebound in PTCs using a comprehensive systems serology approach. We profiled Env-specific antibody characteristics and Fc-effector functions—including antibody-dependent cellular phagocytosis, antibody-dependent neutrophil phagocytosis, antibody-dependent complement deposition, and antibody-dependent NK cell activation—in PTCs and matched non-controllers (NCs). Baseline antibody features prior to ART interruption did not clearly distinguish PTCs from NCs, and antibody responses evolved dynamically and in a highly individualized manner during viral rebound. Notably, distinct and individual-specific shifts in Fc-functional antibody signatures occurred during rebound, indicating that the humoral immune system actively adapts during this critical period. These findings highlight the complexity and adaptability of antibody-mediated responses, shaped by dynamic processes during rebound rather than static baseline features. This work underscores the value of longitudinal immune profiling to delineate how humoral immunity evolves in relation to, but not necessarily driving, post-treatment control.

**IMPORTANCE:**

Most people living with HIV experience a quick return of the virus after stopping antiretroviral therapy (ART). However, a small group—post-treatment controllers (PTCs)—can keep the virus suppressed without ongoing treatment. This study examined whether antibody responses, especially those involving Fc-mediated functions, play a role in this control. This study compared antibody features and immune functions in PTCs and matched individuals who did not control the virus after stopping ART. They found that antibody profiles before treatment interruption did not predict who would control the virus. Instead, antibody responses changed over time in unique ways during viral rebound. These shifts suggest that the immune system adapts dynamically rather than relying on fixed traits. The findings highlight the importance of tracking immune changes over time to better understand how long-term HIV control may occur, even if antibodies alone may not be the driving force behind post-treatment remission.

## INTRODUCTION

During the early stages of HIV infection, a latent viral reservoir is established within CD4+ T cells, which persists despite years of highly effective antiretroviral therapy (ART) (1, 2). Upon interruption of ART, this reservoir can be reactivated to produce virus, leading to viral rebound. However, rare instances of natural control over HIV infection occur in the absence of treatment. Notably, elite controllers (ECs) maintain undetectable viremia without ever receiving ART (3), while post-treatment controllers (PTCs) are able to control viral rebound to low levels after discontinuing ART (4, 5). Defining the mechanisms that underlie these natural forms of viral control can provide valuable insights into the development of novel strategies aimed at achieving a functional cure.

The mechanisms underlying viral control in ECs appear to involve the targeted elimination of infected cells. For instance, certain ECs exhibit robust T-cell responses and rare human leukocyte antigen (HLA) alleles that facilitate cytotoxic T lymphocyte (CTL) responses against conserved HIV peptides presented via major histocompatibility complex (MHC) class I molecules (3). Additionally, polyfunctional antibodies capable of mediating antibody-dependent cellular cytotoxicity against infected cells have been identified (6). Although the precise mechanisms enabling post-treatment control remain poorly understood, emerging evidence suggests that viral control in PTCs may also involve targeted cell elimination through strong HIV-specific CD4+ T-helper responses and natural killer (NK) cell activity (7), as well as a limited size of the viral reservoir both before and during rebound (5, 8). However, since PTCs exhibit considerably weaker HIV-specific CTL responses compared to ECs (5), it is plausible that antibody-mediated mechanisms may play a significant role in viral control.

Antibodies mediate a variety of immunological functions through their Fc region, including recruitment and activation of innate effector cells by engagement of cell-surface Fc-gamma receptors (FcyRs) directly or by binding complement and then engaging complement receptors. These functions play a critical role in antiviral immunity to influenza (9), respiratory syncytial virus (10), SARS-CoV-2 (11), Ebola (12, 13), and others. Recent humoral profiling studies have found that both FcyR3a-mediated NK activity and specific Fc-glycan profiles are associated with delayed rebound after treatment interruption (14). It’s possible that in the absence of a strong CTL response, PTC may be driven by similar antibody-dependent cell-targeting mechanisms.

Additional factors contributing to post-treatment control (PTC) include the size and diversity of the latent viral reservoir, as PTC is more commonly observed in individuals who received treatment during acute primary infection, a period when the reservoir is small and less diverse. However, PTC has also been documented in individuals treated later, during the chronic phase of infection, when a diverse latent reservoir has been established and the humoral response begins to develop broader specificity. Individuals treated during chronic infection also represent the majority of people living with HIV in the world. Because chronically treated individuals possess diverse humoral and viral repertoires, the mechanisms underlying post-treatment control for these individuals potentially differ substantially from individuals treated early during primary infection, as a greater reliance on the breadth and function of the humoral immune response may be more important for chronically treated individuals.

We sought to understand the humoral repertoires of chronically treated PTCs and study-matched non-controllers (NCs) identified in the ontrol of HIV after antiretroviral medication pause (CHAMP) study, which includes longitudinal PTC and NC serum samples from analytical treatment interruption (ATI) studies (15). Using a panel of diverse HIV-1 Envs, we profiled antibody subclass, isotype, and FcyR binding. We also quantified Env-specific antibody effector functions such as antibody-dependent complement deposition (ADCD), antibody-dependent cellular phagocytosis (ADCP), antibody-dependent neutrophil phagocytosis (ADNP), and antibody-dependent NK cell activation (ADNKA). Our results reveal highly variable humoral profiles in both PTCs and NCs prior to ATI. During rebound, these profiles follow individual trajectories that may reflect differences in viral load kinetics.

## RESULTS

### Virological and immunological characteristics

To understand the impact of humoral immunity in chronically treated individuals both before ATI and as viral load evolves after ATI, we focused on individuals with longitudinal samples up to 50 weeks post interruption (10 PTCs, 7 NCs, Table 1). All individuals had undetectable viral loads at baseline (≤2 weeks prior to ATI) except for two PTCs whose viral loads were detectable below 1,000 cp/mL at ATI (321568 and 621422, viral load = 127 cp/mL and 793 cp/mL, respectively, Table 1).

**TABLE 1:** Baseline characteristics of chronically treated individuals

ID	PTC	Parent study	Viral load	CD4	CADNA	CARNA	Post-ATI
			(cp/mL)	(cell/mL)	(cp/10,000)	(cp/10,000)	(days)
44741	Yes	A5068	<50	882	24.95	20.73	0
51270	No	A5170	<50	1,048	24.95	48.19	0
83221	Yes	A5170	<50	987	24.95	20.73	0
83222	No	A5170	<50	1,012	24.95	20.73	−2
83223	No	A5170	<50	1,488	593.23	20.73	−2
83281	Yes	A5170	<50	1,380	51.63	129.99	0
111836	Yes	A5068	<50	885	29.11	20.73	0
212915	No	A5170	<50	561	486.39	619.47	−1
213258	Yes	A5068	<50	904	203.65	37.63	0
220951	Yes	A5170	<50	1,148	ND	ND	−14
251644	No	A5068	<50	691	106.27	173.54	−1
272376	No	A5170	<50	1,246	24.95	20.73	−1
291206	Yes	A5170	<50	688	24.95	57.75	0
291214	No	A5170	<50	1,050	554.9	89.15	−3
321568	Yes	A5068	127	748	ND	ND	0
580615	Yes	A5068	<50	940	24.95	41.55	0
621422	Yes	A5068	793	886	70.01	346.87	0

**Fig 1:**
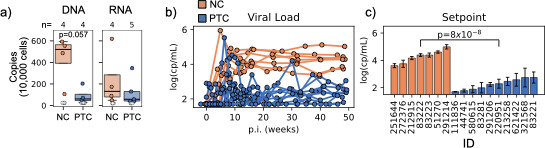
Baseline characteristics. (**a**) Cell-associated DNA and RNA for NC (orange) and PTC (blue). (**b**) Viral load from the time of ART interruption (week 0) through 50 weeks without treatment for NC (orange) and PTC (blue) individuals. (**c**) Viral load setpoints for each NC (orange) and PTC (blue) individual (x-axis) represented as the average and standard deviation of viral load from week 20 to 50. Student’s *t*-test was used to calculate *P* values.

Both PTCs and NCs had similar CD4+ T cell counts at the time of antiretroviral treatment interruption (ATI) (Fig. S1a) suggesting comparable immune states. About half of the NCs (3 of 7) showed the highest levels of cell-associated DNA (Fig. 1a). However, overall levels of cell-associated DNA or RNA did not differ significantly, as several NCs had low or undetectable amounts.

After ATI, all NCs experienced a rapid viral rebound during the first 10–12 weeks, followed by a stable viral set point (Fig. 1b). Rebound viral load profiles among PTCs varied: some individuals reached high viral loads (>10,000 cp/mL) within the first 10 weeks after interruption, while others had much lower levels. Rebound kinetics also reflected this variability, as nearly half of PTCs (4 of 10) maintained <200 cp/mL for longer periods post-ATI, while the rest did not (Fig. S1b). Viral setpoints in PTCs, however, were 100-fold lower on average than the ones in NCs (*P *= 8×10^−8^, *t*-test, Fig. 1c), consistent with viremic post-treatment control.

### Heterogeneous pre-ATI HIV-specific antibody profiles

To map humoral profiles, we measured IgM, IgA, and IgG subclass binding titers to 13 monomeric HIV gp120 Envs, 3 trimeric gp140 Envs, JRFL SOSIP, gp41, and p24 (Table S1). Positivity in antigen-specific binding titers was defined using non-specific binding to albumin as a threshold plus two standard deviations (see Methods). Prior to ATI (baseline), all individuals were positive for total IgG specific to p24 and gp41 (Fig. S2a), while HIV-specific IgA and IgG4 binding titers were not detectable above threshold for any individual (data not shown).

**Fig 2:**
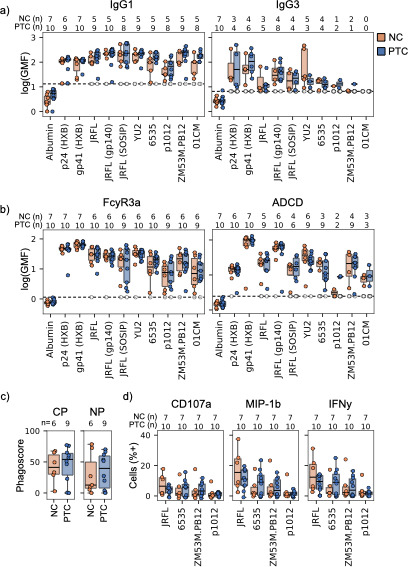
Baseline binding titers and functions. (**a and b**) HIV-specific IgG1, IgG3, and FcyR3a binding titers and C3 deposition. A dashed line indicates the detection threshold defined by non-specific binding to human albumin. Numbers above each graph show the number of NC (orange, total 7) or PTC (blue, total 10) with titers above threshold that were used in statistical comparisons using Mann–Whitney with Bonferroni correction for multiple comparisons. Individuals with titers below the threshold are shown as open circles at the threshold line. HIV antigens are shown across the x-axis. (**c**) ADCP and ADNP phagoscores for NC (orange) and PTC (blue) specific to HIV Env JRFL (gp140). (**d**) HIV Env-specific ADNKA for NC (orange) and PTC (blue) represented as the percent of NK cells induced to express CD107A, IFNy, or MIP-1b for Envs JRFL, 6535, ZM53M, and p1012.

Baseline IgG1 and IgG3 binding titers were not significantly different between PTC and NC (Fig. 2a; Fig. S2b and c). However, we noted a broad heterogeneity in binding titers for PTCs and NCs, where both IgG subtypes varied by up to 50-fold.

To understand whether baseline Fc-mediated effector functions might have an impact on rebound control, we measured HIV-specific antibody binding to FcyRs 2a, 2b, 3a and 3b (Fig. 2b,left and Fig. S3 and b). We found no significant differences in FcyR binding between PTCs and NCs. FcyR binding generally reflected IgG1 breadth as opposed to IgG3, as most individuals had detectable IgG1 binding titers to Envs p1012, ZM53M, and 01 CM but not IgG3. FcyR binding titers were also strongly correlated across FcyRs (Pearson R ≥ 0.93 across all antigens).

To determine whether there were direct functional differences between PTC and NC baseline repertoires, we measured (ADCD, Fig. 2b, right and Fig. S3c), neutrophil and monocyte phagocytosis (ADNP and ADCP, respectively, Fig. 2c), and (ADNKA, Fig. 2d). As with IgG1 and IgG3 titers, HIV-specific ADCD was also heterogeneous for both PTCs and NCs with no significant differences. Similarly, ADCP or ADNP specific to trimeric JRFL gp140 Env (Fig. 2c) did not differ, as both PTCs and NCs varied broadly in phagoscores. HIV-specific ADNKA was measured by degranulation (CD107a), as well as chemokine and cytokine expression (MIP-1b and IFNy) against four HIV Envs (Fig. 2d). We found a high degree of variability among both PTCs and NCs with no significant differences in ADNKA overall.

### Humoral responses during rebound

**Fig 3:**
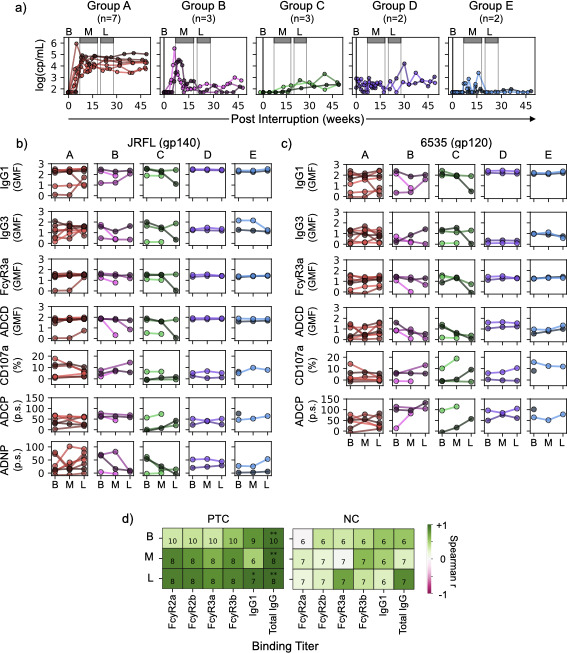
Post-interruption viral load and humoral profiles. (**a**) Post-interruption viral load dynamics for NC (group A, red) and PTC (groups B–E). PTC viral load profiles were clustered into four groups (B–E) based on similarity. Gray boxes span mid (weeks 7–20) and late (weeks 20–28) rebound. (**b**) JRFL Env-specific binding titers for IgG1, IgG3, FcyR3a, C3 deposition, and ADNKA (% CD107a+), ADCP, and ADNP phagoscores (p.s.) for NC group A and PTC groups B–E at baseline (**b**), during mid rebound (M, weeks 7–20) and during late rebound (L, weeks 20–28) for each individual. (**c**) Humoral responses to HIV Env 6535 (represented in the same way as in panel **b**). (**d**) Spearman correlations between p24-specific ADCD and IgG/FcyR binding titers for PTC (left) and NC (right). Negative correlations are shaded pink, and positive correlations are shaded green. The number of data points for each correlation is indicated along with Bonferroni-corrected *P* values (*P ≤ 0.05, **P ≤ 0.005).

We next examined the interplay between viral rebound and humoral responses after ATI. With the samples available, we defined three time periods for which we could compare both virological and humoral data: baseline (B) is prior to ATI, mid-rebound (M) spans weeks 7–18 of ATI, and late rebound (L) includes weeks 20–28 (Fig. 3a B,M, and L labels). To understand how humoral responses are associated with these rebound dynamics, we measured immunoglobulin titers, Fc-receptor binding, and effector function from longitudinal serum samples for each individual through mid and late rebound. Similar to baseline, we found no significant difference in either antibody or FcyR binding titers (Fig. S4 and S5, respectively) or functions (Fig. S6) at mid or late rebound.

We noticed that PTCs experienced a variety of different rebound viral load profiles, suggesting highly individualized viral dynamics. To better describe and interpret humoral responses in light of these diverse rebound viral load kinetics, we classified all individuals based on their post-interruption viral load profiles (groups A–E, Fig. 3a). Group A was composed exclusively of NCs, who all experienced rapid increases in viral load followed by a stable set point. Groups B–E show the different viral load profiles that we observed among PTCs for the first 50 weeks of ATI. Like NCs, group B (*n* = 3) also experienced a rapid increase in viral load during the first 10 weeks of ATI; however, control was established near week 15. Conversely, group C (PTC, *n *= 3) was characterized by a slow but clear increase in viral load. Groups D and E (PTC, *n* = 2 each) both experienced unstable viral load; however, by week 20, group E had established control to undetectable levels, while group D had not. Group D was composed of the only two PTCs in our cohort who had detectable viral loads at baseline (321568 and 621422, Table 1).

Humoral time courses specific to HIV Envs JRFL (gp140) and 6535 (gp120) are shown in Fig. 3b and c, while changes in humoral time courses from baseline to early rebound and early to late rebound for all antigens are shown in Fig. S7 through S9.

Prior to ATI, highly variable IgG1 and IgG3 titers were observed for NCs. This trend continued through early and late rebound (Fig. 3b and c, group A), where some individuals experienced 10- to 100-fold increases or decreases in IgG1 and IgG3 titers specific to JRFL and 6535 Envs. Changes in FcyR binding titers were highly correlated through rebound (data not shown) and tended to reflect the wider breadth of IgG1 as opposed to IgG3 (compare Fig. S4b and c to Fig. S5), similar to baseline Fc-receptor binding (Fig. 2b).

Functional antibody trajectories were variable among NCs, particularly ADCD, where we observed both transient changes specific to early rebound and steady increases through the early and late rebound time points against Env 6535, depending on the individual (Fig. 3c). ADNKA (measured by expression of CD107a) and ADNP, however, did appear to follow a broader trend of decreased NK and increased NP over time. For example, NCs with ≥10% CD107a+ NK cells at baseline experienced a decrease in ADNKA by late rebound (Fig. 3b and c). Conversely, individuals with low ADNP phagoscores (≤50) tended to develop higher ADNP phagoscores by late rebound (Fig. 2B). These trends, however, were not statistically significant.

PTC rebound group B was defined by a rapid increase in viral load during the early post-ATI time point (similar to NCs) followed by a rapid decrease and viremic control by the late post-ATI time points. Similar to NCs, there were no clear rebound-associated trends in IgG1 or IgG3 titers or FcyR binding. For example, one individual in group B experienced a ~100-fold decrease in 6535-specific IgG1 levels at early rebound, while another in group B experienced a nearly 100-fold increase at late rebound (Fig. 3c).

Group C also consisted of largely heterogeneous humoral trajectories. We did observe a possible trend of decreased ADCD for individuals in both groups B and C and, for some individuals, increased ADNKA specific to 6535 (Fig. 3c). Likewise, individuals from both groups tended to experience a loss of JRFL-specific ADNP from baseline to late rebound.

PTC groups D and E shared an initial period of unstable viral load through early post-ATI and then differed in whether this trend continued (group D) or viral load was controlled during late post-ATI time points (group E). Unlike groups A, B, and C, individuals in groups D and E had very stable IgG1/G3 titers, FcyR binding, and ADCD over time. Interestingly, although the individuals had detectable viral load at baseline and greater fluctuations in viral load during rebound, the humoral profiles of group D appeared to be more stable over time than group E (Fig. S7 through S9).

Finally, we examined correlations between humoral functions and binding titers across baseline, mid, and late rebound. We found significant correlations between p24-specific ADCD and total p24-IgG binding titers across all three time points in PTCs but not in NCs (Fig. 3d, Spearman r ≥ 0.915, *P* < 0.005). In PTCs, this was accompanied by correlations between p24-specific FcyR binding titers and ADCD (Spearman 0.3 < r< 0.5).

## DISCUSSION

Here, we aimed to determine whether humoral responses exhibited enhanced Fc-mediated effector functions in PTCs compared to NCs prior to or after ATI. Previous studies have suggested that antibody-dependent functions, but not necessarily titers, are associated with elite control of HIV (6), with PTC in individuals treated during acute infection (16), and with delayed rebound among NCs during ATI (14, 17). To investigate this, we employed a panel of diverse Env variants to capture humoral responses to the diversifying viral reservoir during rebound viremia. Our results reveal substantial heterogeneity in antibody titers, Fc-receptor binding, and Fc-mediated functions, with variability across individuals being more pronounced than differences based on controller status. While all individuals exhibited detectable p24 and gp41-specific IgG, Env-specific humoral signatures varied dramatically both at baseline and during rebound, with variation observed in IgG subtypes. This heterogeneity likely reflects the unique dynamics of rebound viremia occurring in each individual and the diversity of latent viral isolates that are reactivated in the absence of treatment.

We observed that baseline HIV-specific antibody binding titers, FcyR binding, and Fc-mediated effector functions were not associated with post-treatment control (PTC) status, indicating that the humoral functional profiles at the time of treatment interruption do not predict control following ART cessation, at least within this cohort. Previous studies, including our own, have shown that Fc-mediated effector functions, particularly NK cell activity, prior to ART interruption can be linked to delayed viral rebound during ATI (17). However, in that study, antibodies with distinct functional properties also mirrored the transcriptional activity of the viral reservoir, which itself can influence viral rebound dynamics. In contrast, in the present study, PTCs and NCs, although heterogeneous in their baseline reservoir measures, exhibited similar cellular HIV RNA levels. Furthermore, although non-neutralizing antibody functions pre-ART interruption were not enriched or predictive of PTC, it is likely that an individual’s baseline humoral repertoire influences viral rebound by selecting for the replication of viral variants resistant to immune control. In fact, autologous neutralizing antibodies (aNAbs) have been reported to contribute to the control of susceptible reservoir viruses (18), and a stronger aNAb response was reported as a pre-ATI feature distinguishing PTC from NC (19).

Consistent with studies of acutely treated individuals (16, 19), we find that the humoral profiles of chronically treated PTCs and NCs evolve in response to viral replication during rebound. Because viral exposure can drive humoral evolution (16), we stratified the PTCs in our cohort into groups that reflect similar post-ATI viral load profiles in terms of kinetics and stability. These groups were not thought to demonstrate clear biological differences but rather allow to interpret the antibody data based on viral kinetic patterns. Indeed, we found that individuals in PTC groups D and E were characterized by persistent but low-level viremia that was either unstable (group D) or transient (group E), and these individuals had some of the most stable post-ATI humoral profiles in our study, despite the fact that the individuals in group D had detectable viral load at baseline. Conversely, NCs and PTC group B, whose initial viral loads were similarly high had dynamically changing humoral profiles during rebound. PTC group C, however, experienced low-level viremia (similar to group D) but experienced changing humoral profiles like groups A and B. The viral load trajectories of individuals in group C steadily increased, while those of group D were unstable and fluctuated during mid and late rebound. It’s possible that low-level viremia can drive humoral adaptation when exposure is steady and consistent. Future studies are needed to identify humoral signatures associated with viral control by investigating time-dependent correlations between viral load dynamics and changes in humoral features.

The diverse viral load dynamics observed in this small cohort of 10 PTCs suggest that viral control can manifest at varying levels and times during rebound. Although we detected changes in individual humoral profiles during both early and late rebound, these changes did not consistently correlate with viral load dynamics, highlighting the complexity of the relationship between humoral immune responses and viral control.

Interestingly, p24-specific IgG associated with ADCD was highly correlated in PTCs but not NCs. As p24 is an internal capsid protein, these antibodies are unlikely to mediate direct antiviral activity. Their presence may instead reflect immune recognition of viral debris following the lysis of virus-producing cells via other mechanisms (i.e., by cytotoxic T cells or NK cells). Such responses could signify effective immune clearance or low-level antigen exposure. Indeed, previous studies have associated p24-specific IgG1 and ADCP with viral control (20). Future mechanistic studies are needed to define whether these antibodies actively participate in viral control or simply mirror a more contained infection state.

Our study is limited by the small sample size and the variability in sampling time points across individuals from different studies. The sample size was further reduced when individuals were stratified based on their rebound viral load profiles. Additionally, we were unable to profile humoral responses to contemporaneous rebound isolates due to the lack of available viral sequences, which limited our characterization of Env-specific humoral characteristics and breadth.

Overall, our findings demonstrate that both the biophysical and functional humoral profiles of chronically treated PTCs and NCs exhibit considerable diversity prior to treatment interruption, and that the changes occurring during rebound are similarly variable across NCs and certain subgroups of PTCs. Since Env is the most accessible HIV antigen, a more comprehensive understanding of the humoral mechanisms of control will likely necessitate concurrent, detailed characterization of the rebound isolates, including sequencing of integrated Env variants across various tissues and their blood-borne descendants. A more refined approach to categorizing different types of PTCs, based on viral load profiles, could help elucidate the relationships between viral load kinetics, control mechanisms, and the adaptive interplay between the virus and host immune responses.

## MATERIALS AND METHODS

### Samples

Pre-ATI and post-ATI samples were obtained from previously completed ACTG (Advancing Clinical Therapeutics Globally for HIV/AIDS and Other Infections) ATI trials, including ACTG A5068 and A5170 (21, 22). Posttreatment controllers were defined as individuals who remained off ART for ≥24 weeks posttreatment interruption and maintained viral loads ≤ 400 copies/mL for at least two-thirds of the time points as described in the CHAMP study (15), regardless of the viral load at ATI. Viral loads > 400 HIV-1 RNA copies/mL were acceptable if the participant was subsequently able to suppress to ≤400 HIV-1 RNA copies/mL and maintained virologic control through week 24 posttreatment interruption.

### Antibody and Fc-receptor binding titers

Antigen-specific antibody isotypes and FcyR were quantified through a multiplexed Luminex assay, as previously described (23). A panel of HIV antigens (insert antigens and source here) was covalently linked to carboxyl-modified MagPlex microspheres (Luminex) via N-hydroxysuccinimide (NHS)-ester linkages via EDC (Thermo Scientific) and Sulfo-NHS (Thermo Scientific). Serum samples were diluted (dilution here) for isotypes and FcyR binding and incubated with coupled beads in 384-well plates at 850 rpm for 2 h at room temperature. After the formation of immune complexes, microspheres were washed three times in 0.1% bovine serum albumin (BSA) and 0.05% Tween20 (Luminex assay buffer) with an automated plate washer (Tecan). Antibody isotypes were stained with mouse anti-human IgG-PE antibodies (Southern BioTech) for 1 h at room temperature. FcyR proteins were biotinylated (BirA500, Avidity) per manufacturer’s instructions and tagged with streptavidin-PE (Agilent) and incubated for 1 h at room temperature. Plates were washed again, and beads were resuspended and run on flow cytometry instruments (iQue, Intellicyt) to determine the geometric mean fluorescent intensity. All experiments were run in duplicate fashion. Positive reactivity was defined as a signal that was 2 × s.d. over non-specific binding to human albumin.

### Antibody-dependent phagocytosis

ADNP and ADCP assays were performed as previously described (24, 25). HIV antigens listed in Table S1 were purchased from ImmuneTech except for BG505 SOSIP, which was manufactured by the protein core at Duke University. HIV antigens were biotinylated using the EZ-link Sulfo-NHS-LC-LC-Biotin kit (Thermo Fisher) and coupled to fluorescent neutravidin beads (Thermo Fisher). Serum was diluted (1:100) and incubated for 2 h at 37°C, following incubation, unbound antibody was washed. For ADCP, immune complexes were incubated overnight (16–20 h) at 37° with cultured THP-1 (ATCC). For ADNP, primary neutrophils were isolated from whole blood with ammonium–chloride–potassium lysis buffer. Immune complexes and primary neutrophils were incubated for 1 h at 37°C. After incubation, cells were washed, THP-1 (ADCP) was subsequently fixed with 4% paraformaldehyde (PFA), primary neutrophils (ADNP) were stained for CD66b+ marker (BioLegend) and fixed with 4% PFA. Subsequent flow cytometry was performed with an iQue (IntelliCyt). A phagocytosis score for both assays was determined as % cells positive × geometric mean fluorescence intensity of positive cells. All assays were performed in duplicate fashion; for ADNP, two healthy donors were utilized.

### Antibody-dependent NK activation

ADNKA assays were performed as previously described (24). ELISA plates were coated with HIV antigens (insert antigens and source here) at 3 μg/mL and incubated at 37°C for 2 h. Plates were washed and blocked with 5% BSA and incubated at 4°C overnight. Primary NK cells were isolated from buffy coats (MGH) from two healthy donors using the RosetteSep isolation kit (StemCell Technologies). NK cells were incubated overnight with supplemented IL-15 (Miltenyi Biotec) at 37°C. Serum was diluted (1:50) and incubated on ELISA plates for 2 h at 37°C. During incubation, a staining cocktail was prepared with anti-CD107a-PE-Cy5 (BD), brefeldin-A (Sigma), and GolgiStop (BD) and added to NK cells. After the incubation plates were washed, NK cells were added at a concentration of 5 × 10^6^ per well. Serum and NK cells were incubated for 5 h at 37°C. NK cells were subsequently stained with Perm and B (Thermo Fisher) and surface markers were stained with anti-CD16 APC-Cy7 (BD) and anti-CD56-PE-Cy7 (BD). Intracellular markers were stained with anti-IFNy APC (BD) and anti-MIP-1B PE (BD). Subsequent flow cytometry was performed with an iQue (IntelliCyt). NK cells were verified as CD56+, CD16+, and CD3− and activation was determined as the percentage of NK cells CD107a+, IFNy+, or MIP-1B+. Healthy donors were performed in replicate fashion for all assays.

### Antibody-dependent complement deposition

ADCD assays were performed as previously described (26). HIV antigens (insert antigens and source here) were biotinylated using the EZ-link Sulfo-NHS-LC-LC-Biotin kit (Thermo Fisher) and coupled to fluorescent neutravidin beads (Thermo Fisher). Diluted serum (1:10) and coupled beads were incubated at 37°C for 2 h to form immune complexes. Lyophilized guinea pig complement (Cedarlane) was reconstituted with water and added to gelatin veronal buffer containing Mg2+ and Ca2+ (GVB++, Sigma Aldrich). After incubation, plates were washed, and the complement solution was added and incubated for 50 min at 37°C. The reaction was then quenched with two washes with 15 mM EDTA in PBS. Immune complexes were stained with fluorescein-conjugated goat IgG fraction to guinea pig complement C3 (Mp Bio). All experiments were performed in duplicate.

### Data processing and statistics

Luminex and ADCD thresholds of detection were defined by nonspecific binding of samples to human albumin plus two standard deviations. Data below this threshold were excluded from statistical comparisons. Univariate distributions of each feature were confirmed to be non-parametric using Shapiro and Levene tests. Univariate comparisons between PTCs and NCs were performed using Mann-Whitney, and *P* values were corrected for each timepoint and readout group (e.g., *P* values for baseline IgG1 were corrected for all antigens measured) using the Bonferroni method. Correlations were calculated using the Spearman method due to the presence of outlier data points. Spearman correlations were calculated for each humoral function across antigen-matched binding titers (total IgG, IgG1, IgG3, and FcyR2a/2b/3a/3b) and *P* values were corrected using the Bonferroni method.

## Data Availability

All data are presented in the main figures and supplementary figures. Raw flow cytometry and Luminex data are available upon request from the corresponding author.

## SUPPLEMENTAL MATERIAL

Supplemental material jvi.01790-25-s0001.pdf Figures S1 to S9; Table S1.

ASM does not own the copyrights to Supplemental Material that may be linked to, or accessed through, an article. The authors have granted ASM a non-exclusive, world-wide license to publish the Supplemental Material files. Please contact the corresponding author directly for reuse.
