# Supplementary material for: The functional antibody landscape in HIV post-treatment controllers is heterogeneous
Source: J Virol. 2025 Nov 28;99(12):e01790-25. doi: 10.1128/jvi.01790-25 (PMC12724254; doi:10.1128/jvi.01790-25)
Supplement: Supplemental material — Figures S1 to S9; Table S1. [file jvi.01790-25-s0001.pdf]

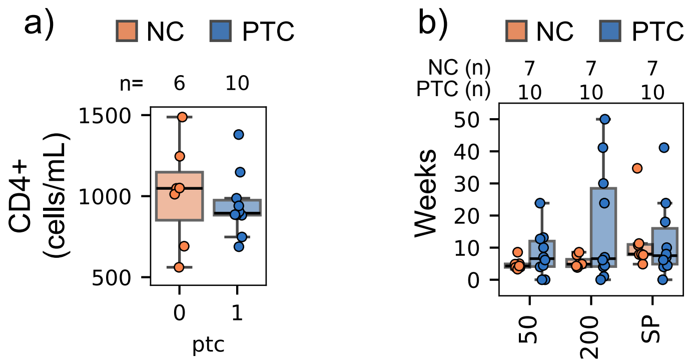

Figure S1. Baseline Immunological Characteristics. (a) Baseline CD4 count for NCs and PTCs. (b) Initial viral load rebound kinetics represented as the time to reach a detectable viral load (50cp/mL), the time to reach 200cp/mL or the time to reach setpoint (SP) after stopping ART for NC (orange) and PTC (blue).

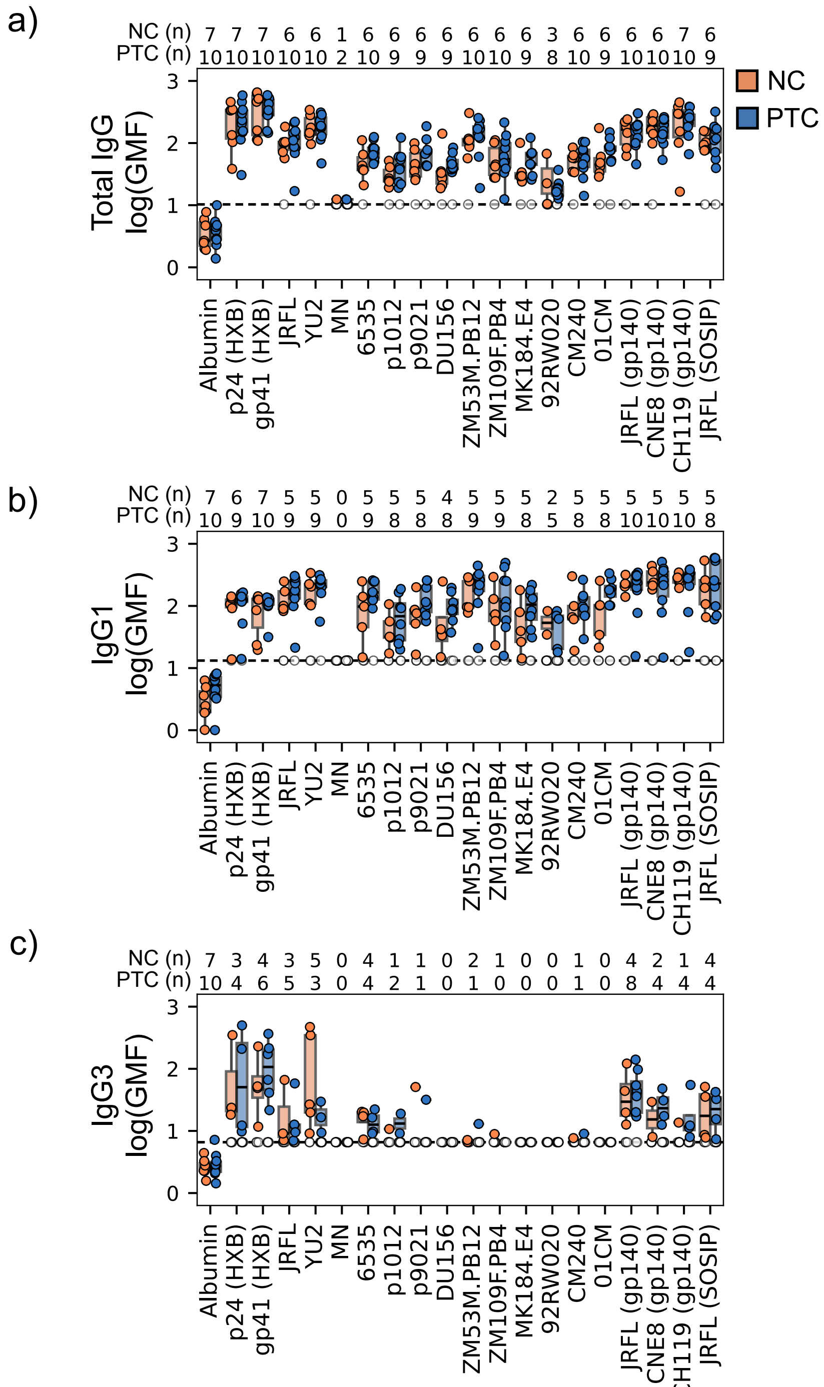

Figure S2. Baseline HIV-Specific Binding Titers. HIV-specific binding titers of total IgG (a), IgG1 (b) and IgG3 (c) isotypes. Dashed line indicates detection threshold defined by non-specific binding to human albumin. Panels above each graph show the number of NC (orange, total 7) or PTC (blue, total 10) with titers above threshold that were used in statistical comparisons (Mann-Whitney). Individuals with titers below threshold are shown as open circles at the threshold line. HIV antigens are shown across x-axis.

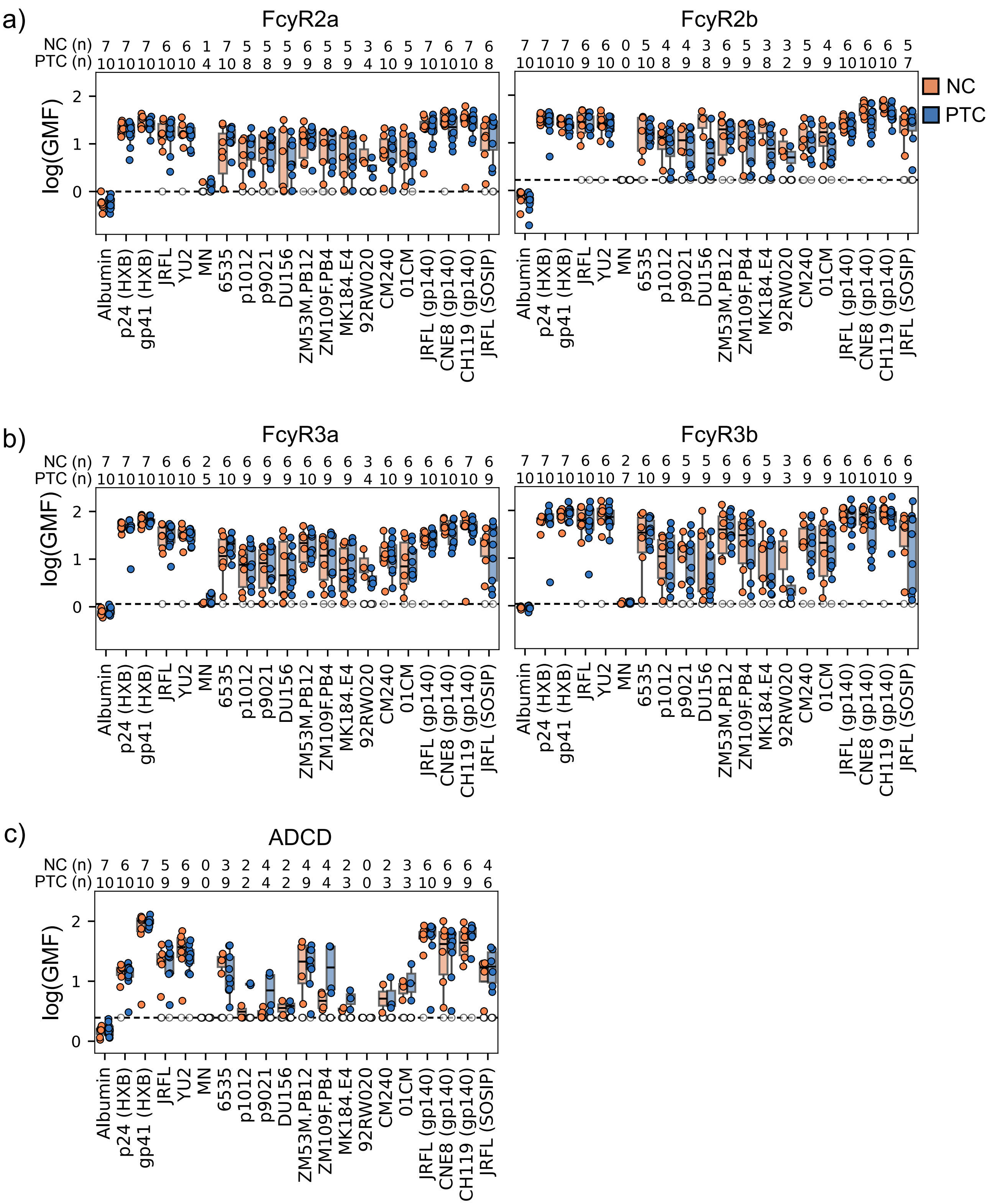

Figure S3. Fc-receptor Binding Titers and C3 Deposition. HIV-specific binding titers and percent reactivity are shown for FcyR2a/2b (a), FcyR3a/3b (b) and C3 deposition (c). Dashed line indicates detection threshold defined by non-specific binding to human albumin. Panels above each graph show the number of NC (orange, total 7) or PTC (blue, total 10) with titers above threshold that were used in statistical comparisons (Mann-Whitney). Individuals with titers below threshold are shown as open circles at the threshold line. HIV antigens are shown across x-axis.

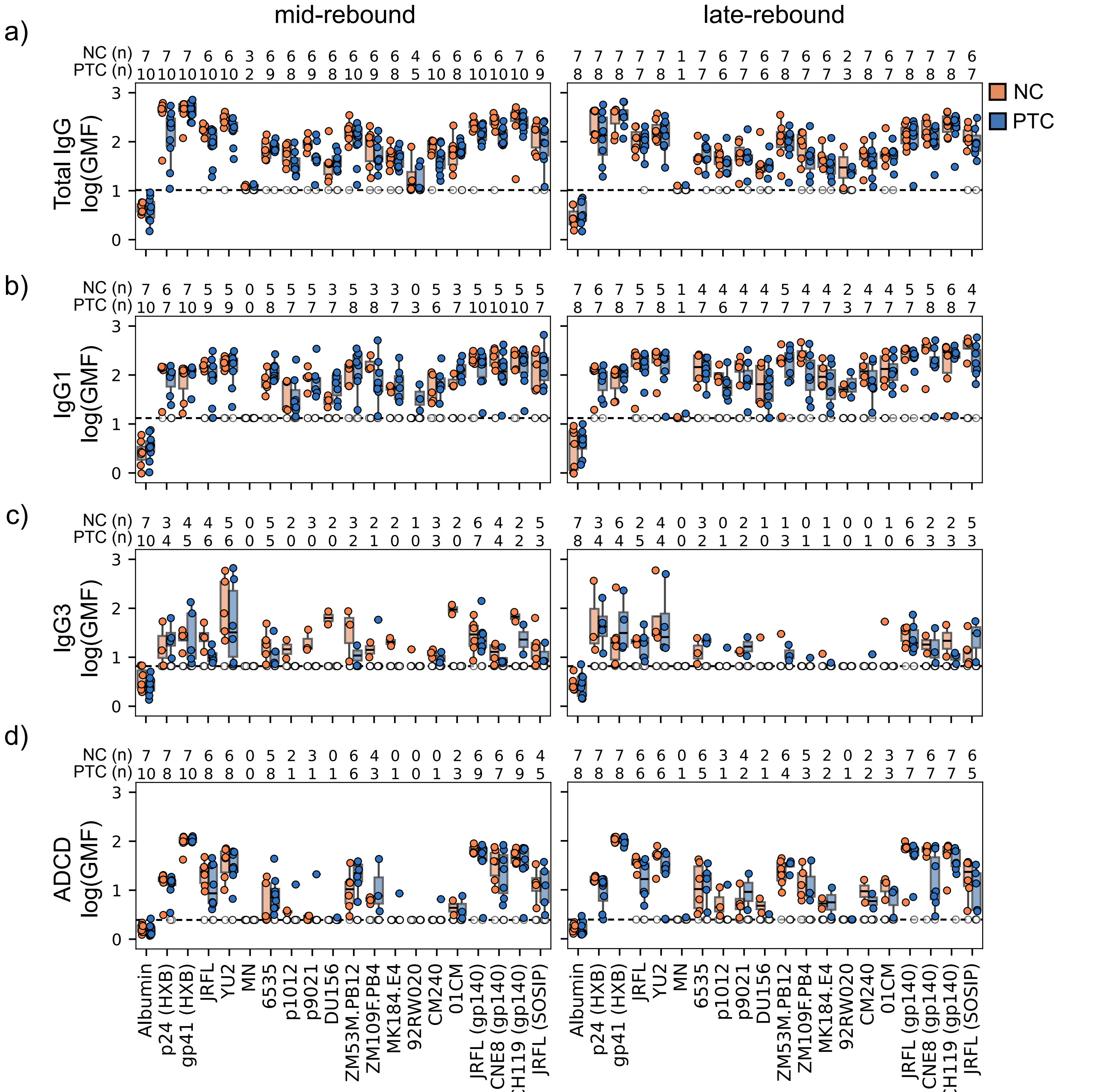

Figure S4. IgG Binding Titers and ADCD Post-ATI. HIV-specific total IgG (a), IgG1 (b) and IgG3 (c) binding titers and ADCD (d) at mid (left) and late (right) rebound. Dashed line indicates detection threshold defined by non-specific binding to human albumin. Panels above each graph show the number of NC (orange, total 7) or PTC (blue, total 10) with titers above threshold that were used in statistical comparisons (Mann-Whitney). Individuals with titers below threshold are shown as open circles at the threshold line. HIV antigens are shown across x-axis.

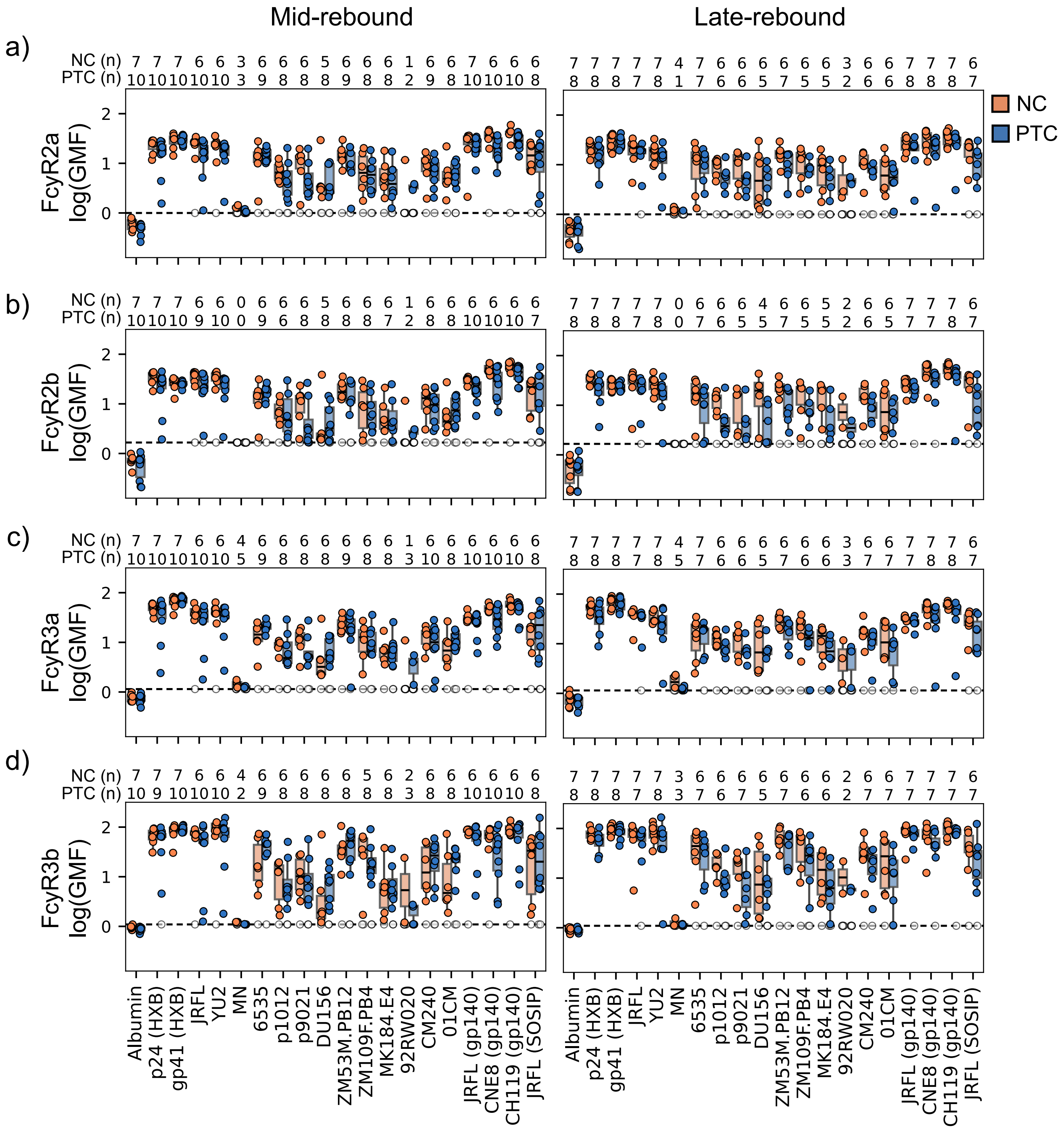

Figure S5. FcyR Binding Titers Post-ATI. HIV-specific binding titers for FcyR2a (a), FcyR2b (b), FcyR3a (c) and FcyR3b (d) at mid (left) and late (right) rebound. Dashed line indicates detection threshold defined by non-specific binding to human albumin. Panels above each graph show the number of NC (orange, total 7) or PTC (blue, total 10) with titers above threshold that were used in statistical comparisons (Mann-Whitney). Individuals with titers below threshold are shown as open circles at the threshold line. HIV antigens are shown across x-axis.

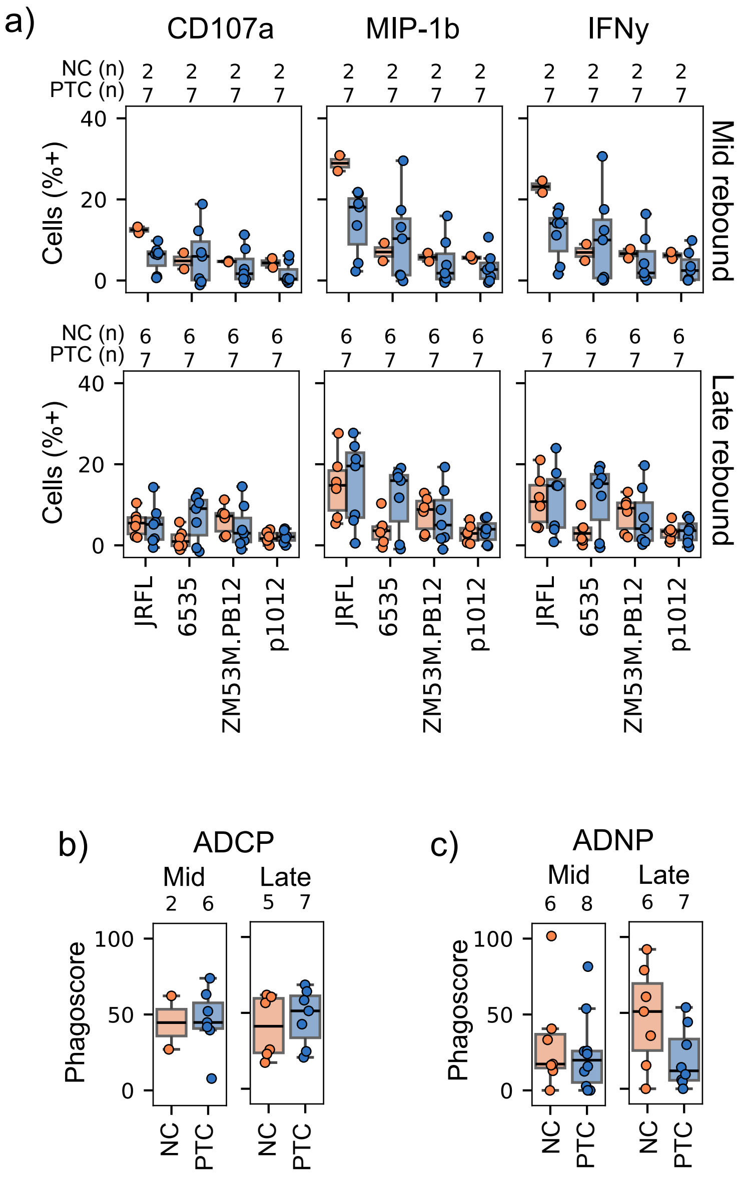

Figure S6. Changes in ADCP, ADNP and ADNKA During Rebound. (a) ADNKA measured in terms of CD107a, MIP-1b and IFNy expression for Mid (top) or Late (bottom) rebound across HIV Envs JRFL, 6535, ZM53M.PB12 and p1012. PTC are shown in blue, NC are orange. Due to sample availability only two data points were collected for NCs for Mid rebound. (b) ADCP and (c) ADNP against JRFL (gp140) for Mid and Late rebound for PTC (blue) and NC (orange). Numbers above each bar indicate the number of data points shown and the number of points used for Mann-Whitney statistical comparisons.

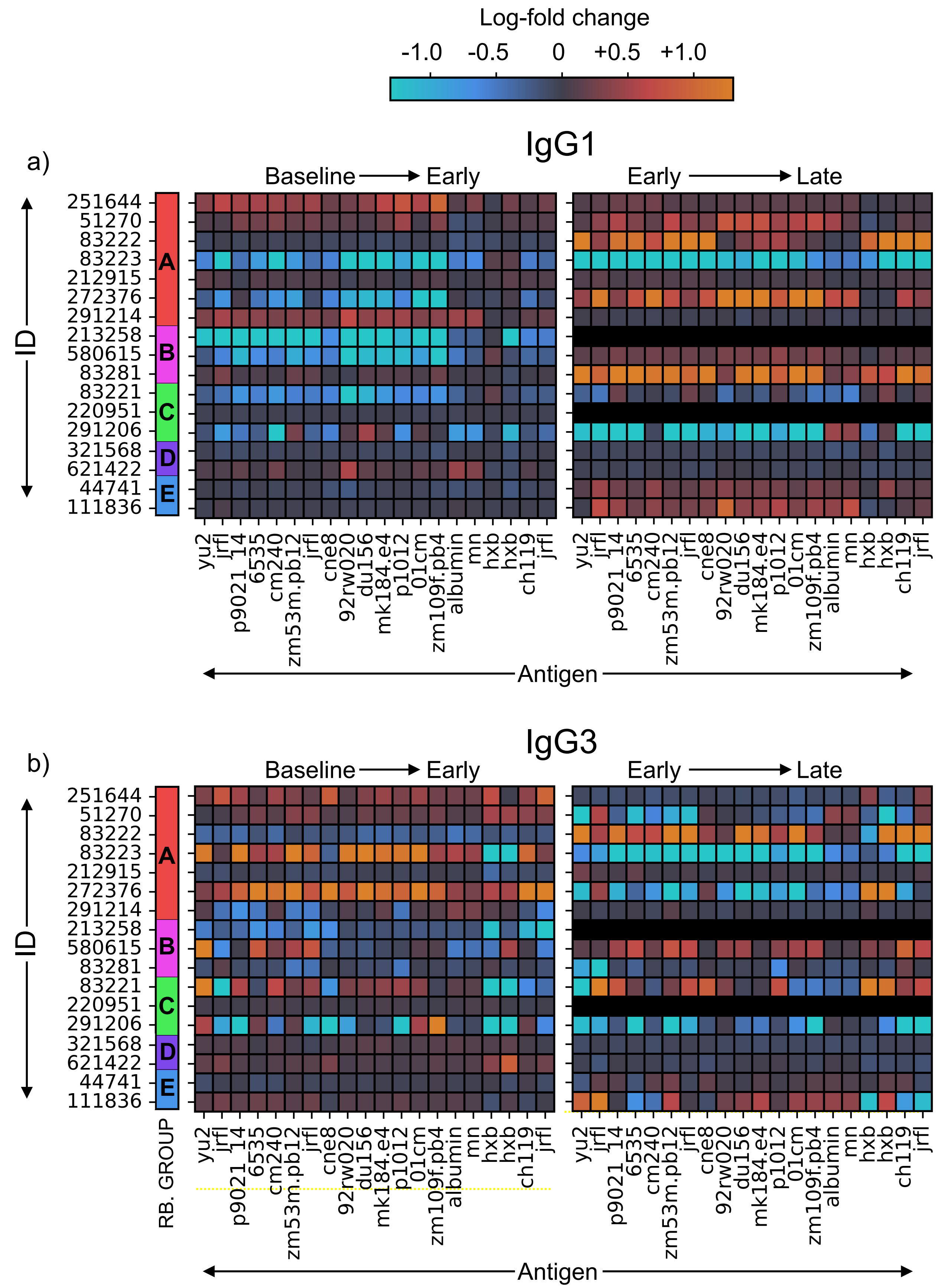

Figure S7. Changes in IgG1 and IgG3 Binding Titers. The log-fold change in binding titer from baseline to early rebound (left) and early rebound to late rebound (right) for each antigen (x-axis) and individual (y-axis) are shown. Individuals are ordered based on rebound group. Values are color coded, where blue indicates up to 10-fold decreases and red indicates up to 10-fold increases.

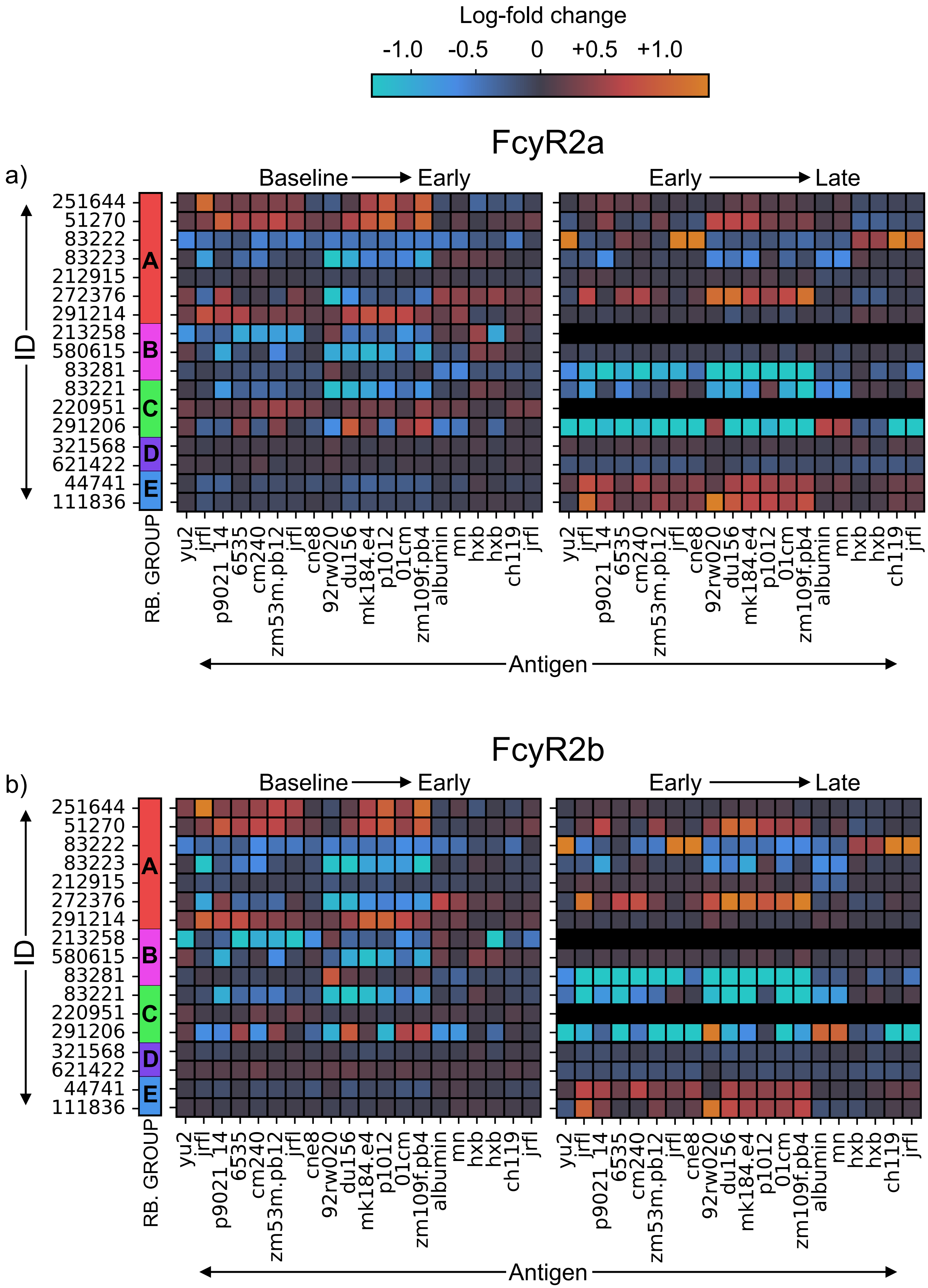

Figure S8. Changes in FcyR2a and FcyR2b Binding Titers. The log-fold change in binding titer from baseline to early rebound (left) and early rebound to late rebound (right) for each antigen (x-axis) and individual (y-axis) are shown. Individuals are ordered based on rebound group. Values are color coded, where blue indicates up to 10-fold decreases and red indicates up to 10-fold increases.

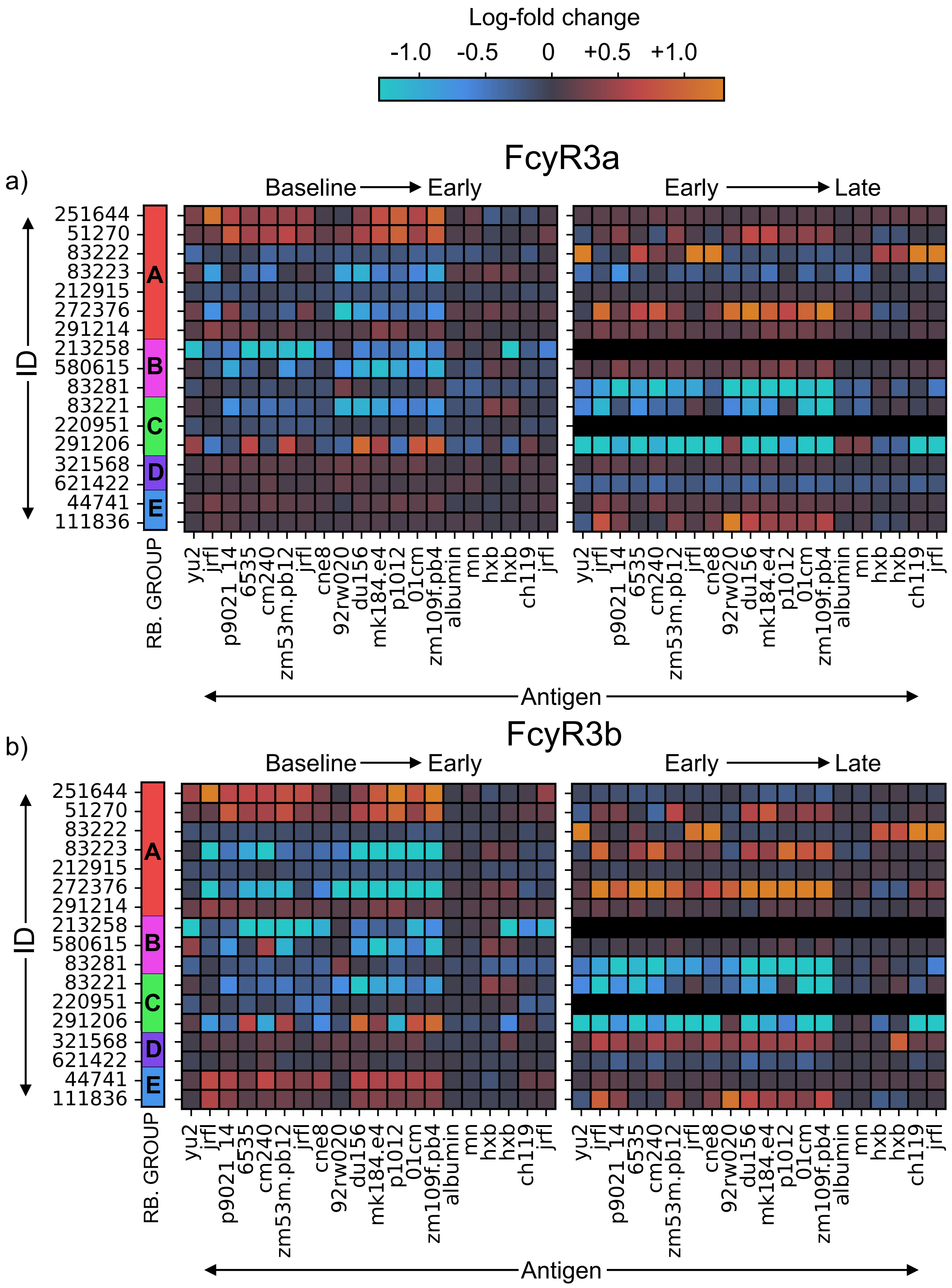

Figure S9. Changes in FcyR3a and FcyR3b Binding Titers. The log-fold change in binding titer from baseline to early rebound (left) and early rebound to late rebound (right) for each antigen (x-axis) and individual (y-axis,) are shown. Individuals are ordered based on rebound group. Values are color coded, where blue indicates up to 10-fold decreases and red indicates up to 10-fold increases.

Table S1. Env antigen characteristics.

isolate	abbrev.	protein	conformation	clade	accession	origin
JRFL	JRFL	gp120	monomeric	B	AAB05604	usa
YU2	YU2	gp120	monomeric	B	P35961	
MN	MN	gp120	monomeric	B	AAC31819	usa
6535	6535	gp120	monomeric	B	AAW64253	usa
p1012.tc21.3257	p1012	gp120	monomeric	B	ABY50633	usa
p9021_14.b2.4571	p9021	gp120	monomeric	B	ABY50691	usa
du156.12	DU156	gp120	monomeric	C	ABD83635	south africa
ZM53M.pb12	ZM53M	gp120	monomeric	C	AAR09394	zambia
ZM109F.pb4	ZM109F	gp120	monomeric	C	AAR09542	zambia
MK184.W0M.ENV.E4	MK184	gp120	monomeric	C/D	ABA61543	kenya
92rw020	92RW020	gp120	monomeric	A	AAT67478	rwanda
cm240	CM240	gp120	monomeric	CRF01_AE	AFJ93254	thailand
01cm_0002bby	01CM	gp120	monomeric	CRF02_AG	AAR21908	cameroon
CNE8	CNE8	gp140	trimeric	CRF01_AE	ADI62622	china
CH119	CH119	gp140	trimeric	CRF07_BC	ABL67458	china
JRFL	JRFL	gp140	trimeric	B	AAB05604	usa
JRFL	JRFL	SOSIP	trimeric	B	AAB05604	usa
